# Oxidative stress-induced inflammation in susceptible airways by anthropogenic aerosol

**DOI:** 10.1371/journal.pone.0233425

**Published:** 2020-11-18

**Authors:** Zaira Leni, Laure Estelle Cassagnes, Kaspar R. Daellenbach, Imad El Haddad, Athanasia Vlachou, Gaelle Uzu, André S. H. Prévôt, Jean-Luc Jaffrezo, Nathalie Baumlin, Matthias Salathe, Urs Baltensperger, Josef Dommen, Marianne Geiser

**Affiliations:** 1 Institute of Anatomy, University of Bern, Bern, Switzerland; 2 Laboratory of Atmospheric Chemistry, Paul Scherrer Institute, Villigen, Switzerland; 3 Univ. Grenoble Alpes, CNRS, IRD, INP, IGE, Grenoble, France; 4 Department of Internal Medicine, University of Kansas Medical Center, Kansas City, KS, United States of America; University of Nebraska-Lincoln, UNITED STATES

## Abstract

Ambient air pollution is one of the leading five health risks worldwide. One of the most harmful air pollutants is particulate matter (PM), which has different physical characteristics (particle size and number, surface area and morphology) and a highly complex and variable chemical composition. Our goal was first to comparatively assess the effects of exposure to PM regarding cytotoxicity, release of pro-inflammatory mediators and gene expression in human bronchial epithelia (HBE) reflecting normal and compromised health status. Second, we aimed at evaluating the impact of various PM components from anthropogenic and biogenic sources on the cellular responses. Air-liquid interface (ALI) cultures of fully differentiated HBE derived from normal and cystic fibrosis (CF) donor lungs were exposed at the apical cell surface to water-soluble PM filter extracts for 4 h. The particle dose deposited on cells was 0.9–2.5 and 8.8–25.4 μg per cm^2^ of cell culture area for low and high PM doses, respectively. Both normal and CF HBE show a clear dose-response relationship with increasing cytotoxicity at higher PM concentrations. The concurrently enhanced release of pro-inflammatory mediators at higher PM exposure levels links cytotoxicity to inflammatory processes. Further, the PM exposure deregulates genes involved in oxidative stress and inflammatory pathways leading to an imbalance of the antioxidant system. Moreover, we identify compromised defense against PM in CF epithelia promoting exacerbation and aggravation of disease. We also demonstrate that the adverse health outcome induced by PM exposure in normal and particularly in susceptible bronchial epithelia is magnified by anthropogenic PM components. Thus, including health-relevant PM components in regulatory guidelines will result in substantial human health benefits and improve protection of the vulnerable population.

## Introduction

Ambient air pollution has negative impacts on human health resulting in more than 4.5 million premature deaths each year [1–3]. Epidemiological studies have found consistent relations between morbidity and mortality and mass concentration of particulate matter (PM) with aerodynamic diameters ≤ 2.5 μm (PM_2.5_) or ≤ 10 μm (PM_10_) [4].

Up to date, PM mass concentration has been considered as the only appropriate metric to describe its impact on health [4]. Thus, this metric has been used to assess the global burden of premature deaths due to PM [1]. Recent studies, however, revealed associations between health effects and sources and composition of aerosols [5, 6]. PM consists of the carbonaceous fractions, which are primarily secondary organic aerosol (SOA) and elemental carbon, of the secondary inorganic aerosol (SIA) including nitrate, sulfate and ammonium, as well as of metals, soil dust, and sea salt [5]. Although exposure to PM has been linked to various adverse effects, there are only few reports on the role of each PM component or a mixture thereof in pathogenesis [7]. At present, there are increasing indications that anthropogenic components of PM, particularly transition metals and SOA, are of particular concern due to their ability to induce oxidative stress through the generation of reactive oxygen species (ROS) [8–11]. ROS generation has been previously identified as a major mechanism underlying the toxicity of air pollutants by triggering multiple redox-sensitive signaling pathways [12]. It has also been suggested that PM transition metals and organic species (e.g. quinones) lead to the generation of free radicals in the lung environment and thereby induce oxidative stress [13, 14]. The ability of PM from both primary and secondary sources to generate oxidative stress has been described as oxidative potential (OP). Hence, organic carbon and transition-metal particles are of particular importance, due to their ability to promote inflammation [8, 9, 15]. Up to date, knowledge is lacking regarding the impact of PM from different sources on acute responses of the primary target tissue of inhaled particles, i.e., the highly specialized, multifunctional respiratory epithelium [15]. Therefore, it is important to identify PM components that lead to an OP responsible for impairing human health.

We assessed the effects of exposure to PM with different physical characteristics and chemical composition with respect to cytotoxicity, release of pro-inflammatory mediators and gene expression on air-liquid interface (ALI) cultures of normal and diseased, i.e., cystic fibrosis (CF) human bronchial epithelia (HBE). This advanced in-vitro model mimics in-vivo characteristics, thus providing the archetypes of normal and susceptible respiratory epithelia, enabling experimental studies impossible in humans, primarily for ethical reasons. In addition, we directly evaluated the oxidative potential (OP) of PM using common acellular assays [15, 16].

## Materials and methods

### Aerosol sampling and sampling sites

In the present study, we assessed ambient particulate matter (PM) collected on filters from urban or rural sites in Switzerland during winter (January to March) and summer (July to August), reflecting the compositional complexity of PM. The PM_10_ and PM_2.5_ were collected within the Swiss National Air Pollution Monitoring Network (NABEL) onto quartz fiber filters (14.7 cm exposed diameter), using a high volume sampler (500 L/min, Digitel, Volketswil, Switzerland). The samples were 24-h integrated and collected every fourth day in 2013 (Bern PM_10_ and Bern PM_2.5_; sampling site: Bern-Bollwerk E 7° 26.452, N 46° 57.059), or 2013 and 2014 (Magadino PM_10_; sampling site: Magadino-Cadenazzo E 9° 0.735, N 46° 9.556). PM was determined gravimetrically by weighing the filters before and after exposure at a relative humidity (RH) of 50% and a temperature of 20°C after conditioning for 48 h. The uncertainty on PM mass is ~ 15%. All filter samples including the field blanks (unexposed quartz fiber filters) were wrapped in lint-free paper and stored at -18°C before further use. Magadino is located in an Alpine valley in the Southern part of Switzerland and the sampling station classifies as a rural background site. Bern is located on a plateau north of the Alpine crest with the sampling station situated in a street canyon and, thus, classifies as a traffic-influenced site. The average seasonal ambient particle concentrations at the urban roadside were 38 and 21 μg/m^3^ for PM_10_ and 28 and 14 μg/m^3^ for PM_2.5_, for winter and summer, respectively. Thus, in winter 74% and in summer 63% of PM_10_ appeared in the PM_2.5_ size fraction. At the rural alpine valley site, particle concentrations were 27 and 13 μg/m^3^ for PM_10_, winter and summer, respectively, in line with continuous measurements at these sites [17]. While the mass concentrations at the rural site are only 30–40% lower, the main difference between the sites is the composition of PM, with a high contribution of biomass burning in Magadino during winter and a high contribution of vehicular emissions in Bern.

### Preparation of water-soluble filter extracts for cell exposure

Filter punches (diameter 10 mm) were taken and weighted to determine the PM concentration before each cell exposure. Punches from different filters from winter or summer were pooled to form a seasonal composite and to avoid day-to-day variation due to traffic and meteorological conditions. For the field blanks, punches of the same filter surface area were pooled. On the day of cell exposure, punches were allowed to equilibrate for 60 min at room temperature before use, to avoid condensation of ambient humidity. Before cell exposure, filter punches were placed in a 14-mL sterile tube containing 7 mL ultrapure water (18.2 MΩ cm MilliQ water) and incubated for 15 min in a warm (30°C) water bath. Samples were thereafter homogenized by vortexing for 1 min and filtered through a nylon membrane syringe filter (0.45 μm mesh size; Infochroma AG, Zug, Switzerland) to remove insoluble material from the sampling filter as well as the insoluble fraction of the aerosol, i.e. elemental carbon, insoluble organic aerosol (OA) and crustal material [18, 19]. These filters are made to maximize the extraction efficiency for the soluble species, while maintaining compositional integrity, so that the final extraction solution yields sufficient PM_10_ and PM_2.5_ mass that remains representative of the ambient composition. In this study, the yield of PM after filtration compared to total PM was not analyzed. However, we estimate that ~ 80% of the PM_2.5_ is extracted and recovered [19, 20]. This includes all the inorganic ions (contributing ~ 50% of the total mass), most of the organic aerosol (extraction efficiency ~ 70%) and the water-soluble elements (varying according to the site and the element from 15 to 100%).

### Cell cultures and exposures

Cultures of primary human bronchial epithelia (HBE) at the air-liquid interface (ALI) reproduce many features of the native respiratory epithelium like the pseudostratified morphology, distinct apical and basolateral secretomes, epithelial barrier function and mucociliary clearance [20–23]. HBE were isolated from donor lungs. Two normal lungs deemed not suitable for transplantation were obtained from the Life Alliance Organ Recovery Agency (LAORA) of the University of Miami, Miami, FL, USA. A cystic fibrosis (CF) lung, homozygous for the DF508 mutation, was donated by a transplant recipient. Appropriate consents, approved by the Institutional Review Board of the University of Miami, Miami, FL, USA, and conforming to the declaration of Helsinki, were used to obtain all lungs. Cells were collected from proximal conducting airways according to approved protocols [24, 25]. ALI cultures of re-differentiated HBE from all organ donors were evaluated for morphological and functional integrity and pre-phenotyped with respect to cytotoxicity and cytokine release before they were used in the experiments, as previously described [23].

Fully differentiated HBE were exposed at the apical cell surface to the water-soluble PM filter extracts for 4 h. Thereafter, the apical cell surface was washed with phosphate-buffered saline (PBS) to remove the particles, mimicking the defense mechanism of the respiratory epithelium. Control cell cultures were either exposed to extracts from field blanks (filters treated the same way apart from exposure to ambient air), or were left untreated in the incubator. As a positive control and to check for the (pro-)inflammatory response capacity, additional cell cultures were exposed to the bacterial endotoxin lipopolysaccharide (LPS) from *Escherichia coli* (Sigma Aldrich, Buchs, Switzerland) at 10 μg/mL in PBS for 4 h. Three independent experiments with triplicate HBE cultures in each experiment were performed.

### Cell analyses

#### Cytotoxicity

Induction of cell death was evaluated by measuring the release of cytosolic lactate dehydrogenase (LDH) from damaged cells into the apical compartment. Apical washes were collected 4 h and 24 h post exposure and stored at 4°C until analysis using the colorimetric cytotoxicity detection kit^**PLUS**^ (Roche Diagnostics AG, Rotkreuz, Switzerland) according to the manufacturer’s instructions. Maximum release of LDH was estimated in the supernatants of unexposed HBE lysed with 100 μL 1% Triton-X solution for 10 min at 37°C. Cytotoxicity is presented as percentage of maximum LDH release.

#### (Pro-)inflammatory mediators

The release of the (pro-)inflammatory mediators interleukin (IL)-6 and IL-8 was assessed in the basolateral compartment collected at 24 h after exposure to PM filter extracts or to the positive control compound LPS, using the Bio-Plex multiplex bead-based suspension array system and the appropriate detection kit (Bio-Rad Laboratories AG, Cressier, Switzerland) according to the manufacturer’s protocol.

#### Gene expression

We screened 20 genes to evaluate alterations in signaling pathways related to oxidative stress using Gene globe arrays. Gene expression in HBE was examined by isolation of total RNA followed by quantitative real-time polymerase chain reaction (RT-qPCR). Briefly, cells were lysed with TRIzol reagent (Invitrogen) and stored at -80°C until further processed. Isolated RNA (500 ng) was reverse transcribed into cDNA using the QuantiTect reverse transcription kit (Qiagen) following the manufacturer’s recommendations. Real-time PCR was performed in a reaction volume of 25 μL using the QuantiTect SYBR Green PCR kit (Qiagen) and the QuantiTect Primer Assays (Qiagen), amplifying a total of 25 ng cDNA of each sample. Real-time PCR was performed using the Applied Biosystems 7900HT-Fast Real-Time PCR System with a 15-min initial activation step at 95°C and 40 cycles with 15 s denaturation at 94°C, 30 s annealing at 55°C and 30 s extension at 72°C. Subsequently, a melting curve was performed to exclude primer-dimer artefacts and to ensure reaction specificity. Data were analyzed using the RQ Manager 1.2 software. The unsupervised heat map of the entire dataset, clustered based on the Euclidean distance, allowed evaluating the deregulation of specific genes in HBE (see S1 Fig). The deregulation of the gene pattern was confirmed after classification according to PM category (PM_10_, PM_2.5_) and collection site.

### Offline aerosol mass spectrometry (AMS) and organic aerosol (OA) source apportionment

Following the offline AMS technique thoroughly described by Daellenbach et al. [19], four filter punches of 16 mm diameter each were extracted in 15 mL of ultrapure water (18.2 MΩ cm at 25°C with total organic carbon < 3 ppb). The liquid extracts were ultra-sonicated for 20 min at 30°C, then vortexed for 1 min and finally filtered through a nylon membrane syringe of 0.45 μm pore size (see also method section). The resulting solutions were inserted into an Apex Q nebulizer (Elemental Scientific Inc., Omaha, NE, USA) operating at 60°C. Aerosols generated in argon (≥ 99.998% Vol., Carbagas, Gümligen, Switzerland) were dried (Nafion dryer) and directed into a high resolution time-of-flight AMS (HR-ToF-AMS, Aerodyne Research, Inc., Billerica, MA, USA). Data processing was performed with the use of the Source finder toolkit (SoFi version 4.9) [26] for IGOR Pro software (Wavemetrics Inc., Portland, OR, USA). The organic mass spectra obtained by the AMS were analyzed by positive matrix factorization (PMF) with the use of Multilinear engine 2 [27]. PMF application on organic mass spectra measured in Switzerland can be found in the literature [19, 28], while the specific dataset is described in detail by Daellenbach et al. [29]. Briefly, PMF was used to decompose the input water soluble aerosol organic mass spectra (represented as 2D matrix **X**) into factor concentration time series (2D matrix **G**) and factor profiles (2D matrix **F**) by iteratively solving the bilinear equation **X = GF + E**, where the 2D matrix **E** represents the residual matrix. The solution that includes the optimum number of factors (in other words sources) is defined by: (i) the minimization of the sum of the squared residuals weighted by their respective uncertainties and (ii) correlations between factors and externally measured source specific compounds. To assess random errors as well as the robustness of the PMF solution, we adopted the bootstrap analysis, based on random resampling. The water soluble OA was quantified by using the externally defined OC (by Sunset OC/EC analyzer [30] with the EUSAAR2 protocol [31]) and the water soluble OC (by a total organic carbon analyzer [32]) concentrations. Finally, the water soluble OA was scaled to its total OA concentration with the use of recoveries [19].

### Chemical analyses

#### Ion Chromatography (IC)

Soluble anions and cations (K^+^, Na^+^, Mg^2+^, Ca^2+^, NH_4_^+^, Cl^-^, NO_3_^-^, SO_4_^2-^), as well as methane sulfonic acid were analyzed after ultrapure water extraction under mechanical agitation (30 min) by ion chromatography (IC, Dionex ICS3000) on the same extracts. AS/AG 11HC and CS/CG 12A columns were used for anion and cation analyses, respectively, following the protocol from Jaffrezo et al. [33]. The anhydrous sugars levoglucosan, mannosan and galactosan were analyzed using a high-performance anion exchange chromatograph with pulsed amperometric detection [34].

#### Inductively coupled plasma mass spectrometry (ICP-MS)

An ELAN 6100 DRC II PerkinElmer or a NEXION PerkinElmer ICP-MS was used for the analysis of 15 trace elements (Al, Fe, Ti, As, Cd, Cu, Mn, Mo, Ni, Pb, Rb, Sb, Se, V, Zn). Briefly, samples from filter punches were mineralized before analysis, using 5 mL of HNO_3_ (70%) and 1.25 mL of H_2_O_2_, during 30 min at 180°C in a microwave oven (microwave MARS 6, CEM) [34] and filtered.

### Oxidative Potential (OP)

For OP evaluation, PM was extracted in simulated lung fluid (SLF) at iso-concentration (25 μg/mL) and subsequently analyzed by three different chemical acellular assays (dithiothreitol: DTT, 2’7’-dichlorofluorescin: DCFH, ascorbic acid: AA) to assess the intrinsic capacity of PM to oxidize a biological fluid. They all rely on the kinetic depletion of an anti-oxidant (AA) or surrogates (DTT, DCFH), when in contact with the extracted solution of PM. The PM solutions were not filtered to also allow surface reactivity of the PM. The depletion was followed during 30 min with a plate-reader using the absorbance mode at 265 nm for the AA assay, at 412 nm for the DTT assay (titration of DTT by 5,5-dithio-bis-(2-nitrobenzoic acid) DTNB) according to Calas et al. [35]. The oxidation of DCFH into the fluorescent compound DCF was assessed by fluorescence at 530 nm for emission (excitation at 485 nm) according to Foucaud et al. [36]. Samples were corrected for the values of the field blanks and for intrinsic absorbance of the particles present in the sample wells.

### Statistical analysis

All biological data are expressed as mean values ± standard deviation. Statistical analyses were performed using GraphPad Prism 7.00 (GraphPad Software Inc., San Diego, CA). For cytotoxicity and release of inflammatory mediators, the arithmetic mean values from the triplicate cell cultures of each experiment were compared to the mean values of the untreated control cultures by one-way analysis of variance (ANOVA) followed by Dunnett’s *t*-test to compare the treated to the control group, or the Bonferroni test for multiple comparisons. To assess the correlation between IL release and OP, metal or inorganic salts composition, non-parametric Spearman correlation (*r*_*s*_) and statistical significance were calculated using the Student’s *t*-test. A value of *p* < 0.05 was considered statistically significant.

## Results

### Deposited particle dose

The particle dose deposited on cells was 0.9–2.5 and 8.8–25.4 μg per cm^2^ of cell culture area for low and high PM doses, respectively (Table 1). The particles were deposited as bolus and left to interact for 4 h with the respiratory epithelium, where antioxidant defense and mucociliary transport replicate the defense mechanisms. In addition, the removal of the particles after 4 h by washing the apical surface mimics the average residence time of particles deposited in this lung compartment. Thus, the dose deposited reflects the effective dose over this time period. To roughly estimate how the experimentally deposited doses translate to real exposure conditions, the Multiple Path Particle Dosimetry model (MPPD v 3.04) [37] was used (S1 Table), which provides the dose the human tracheobronchial tract can acquire over 24 h. The result of such an approximation shows the high and low doses applied in this study to correspond to a deposited dose in the human tracheobronchial tract of days up to weeks in highly polluted areas reaching 1000 μg/m^3^_,_ PM_2.5_, e.g. China [38], and of several months to years in urban areas in Europe, with typical concentrations of 20 μg/m^3^ [39].

**Table 1:** Effective PM mass deposited on cells.

Specification of PM		Average PM mass deposited on cells, μg per cm^2^ cell culture area	
PM type	Season	Low	High
**Urban roadside PM_10_**			
	Winter	2.54	25.38
	Summer	1.47	14.74
**Urban roadside PM_2.5_**			
	Winter	1.88	18.80
	Summer	0.93	9.30
**Rural alpine valley PM_10_**			
	Winter	1.63	16.28
	Summer	0.88	8.84

### Cellular responses to PM exposure

#### Cytotoxicity

**Fig 1:**
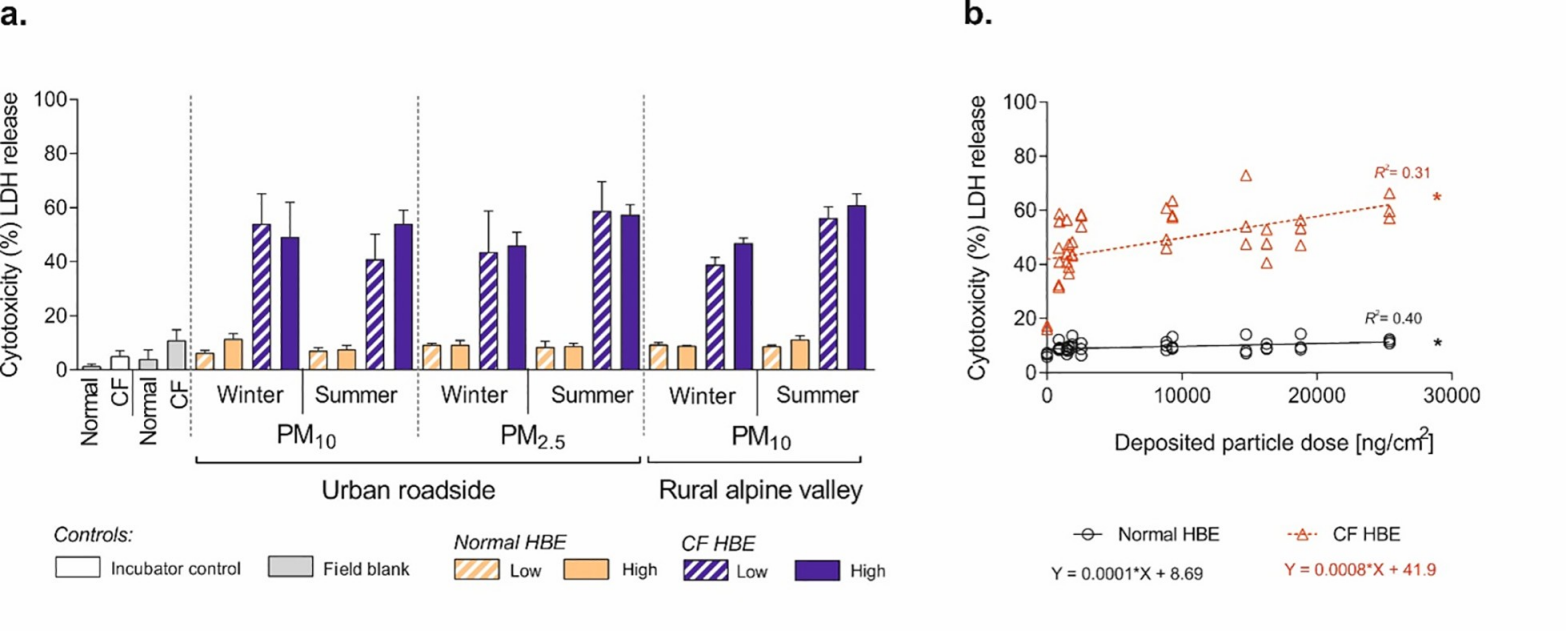
Cytotoxicity in normal and cystic fibrosis (CF) human bronchial epithelia (HBE) 24 h after exposure to seasonally sampled PM from either an urban roadside (Bern) or a rural alpine valley (Magadino). (**a**) Cytotoxicity presented as percentage of total lactate dehydrogenase (LDH) released from damaged cells into the apical compartment. Low dose deposited on HBE = 0.9–2.5 μg per cm^2^ of cell culture area, high dose = 8.8–25.4 μg/cm^2^. The data are presented as mean values and error bars representing 1 standard deviation (SD) of three independent experiments with triplicate cell cultures (total *n* = 9 cell cultures). ANOVA, Dunnett’s multiple comparison). (**b**) Scatter plot and corresponding regression line for normal and CF HBE, evaluating the relationship of cytotoxicity and deposited particle dose (repeated measurements at the same dose are displayed for visualization). * *p-value* < 0.05 compared to filter blank. **Abbreviation:** R^2^ = R-square linear coefficient of determination.

While epithelial morphology did not change upon exposure to PM (data not shown), we found a significant, dose-dependent increase of cytotoxicity. This is reflected by enhanced levels of the cytoplasmic enzyme lactate dehydrogenase (LDH) released from damaged cells into the apical compartment, on average 2.2 fold in normal (*p-value* = 0.0395) and 4.6 fold in CF HBE (*p-value* = 0.0289), compared to field blank controls (Fig 1A and Table 2).

**Table 2:** Cytotoxicity in normal and cystic fibrosis (CF) human bronchial epithelia (HBE) after exposure to PM.

PM, cell models, PM collection, deposited particle dose				LDH release, fold change to field blank controls
PM type	HBE	Season	Dose	
**Urban roadside PM_10_**	**Normal**	Winter	Low / High	1.6 / 2.8
		Summer	Low / High	1.7 / 1.9
	**CF**	Winter	Low / High	4.9 / 4.5
		Summer	Low / High	3.7 / 4.9
**Urban roadside PM_2.5_**	**Normal**	Winter	Low / High	2.3 / 2.3
		Summer	Low / High	2.1 / 2.2
	**CF**	Winter	Low / High	4.0 / 4.2
		Summer	Low / High	5.3 / 5.2
**Rural alpine valley PM_10_**	**Normal**	Winter	Low / High	2.3 / 2.2
		Summer	Low / High	2.2 / 2.7
	**CF**	Winter	Low / High	3.5 / 4.3
		Summer	Low / High	5.1 / 5.5

In addition, Fig 1B shows a positive linear correlation between cytotoxicity and the deposited dose (R^2^ = 0.4011 and 0.3148 for normal and CF HBE, respectively). The increase of cytotoxicity within the same cell model (normal or CF HBE) was independent of seasonal PM collection as well as of sample size fractions, i.e. the coarse PM_10_ and fine PM_2.5_ fractions.

#### Pro-inflammatory mediators

**Fig 2:**
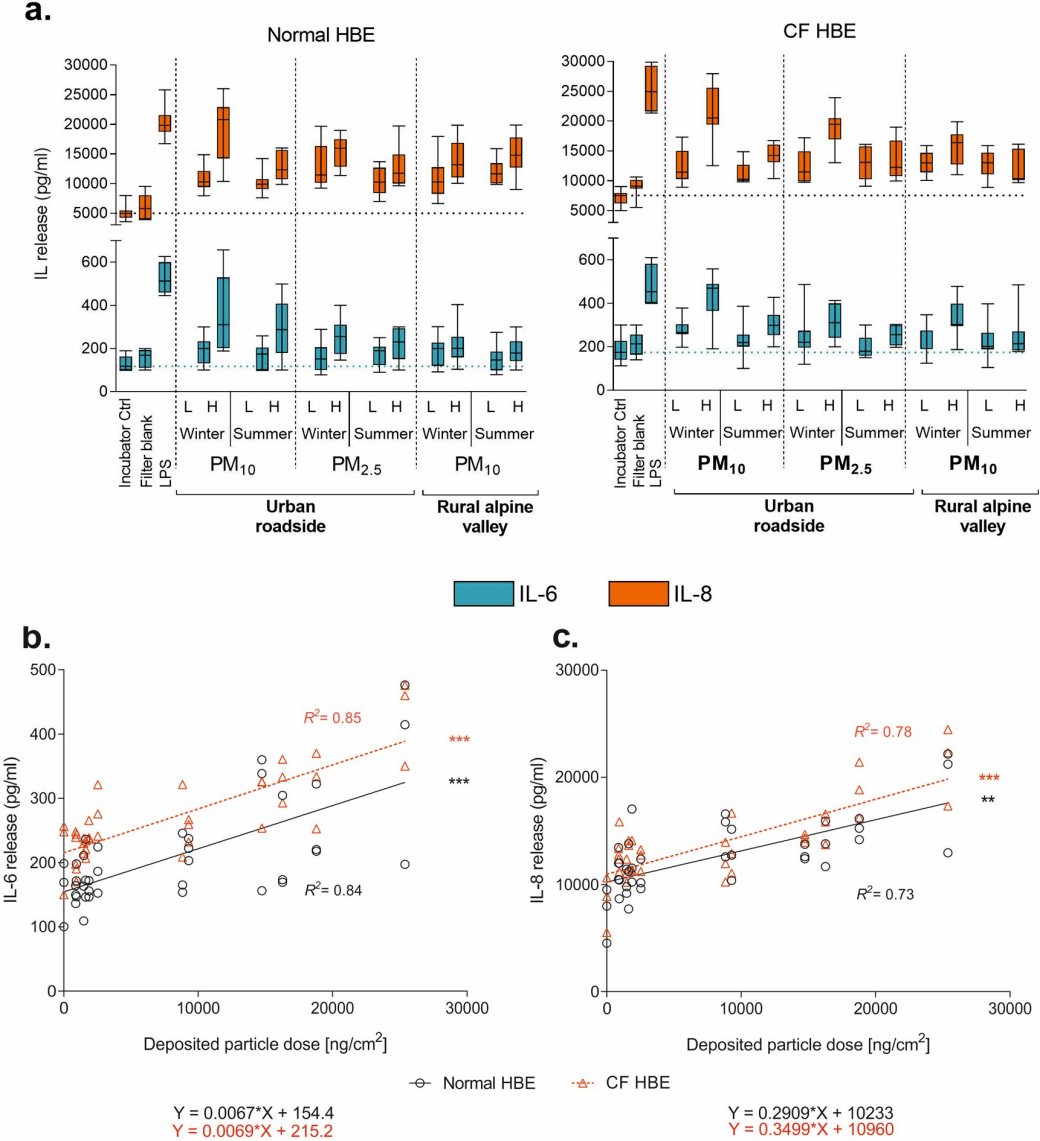
Inflammatory response of normal and cystic fibrosis (CF) human bronchial epithelia (HBE) 24 h after exposure to seasonally sampled PM from either an urban roadside (Bern) or a rural alpine valley site (Magadino). (**a**) Release of interleukin (IL)-6 and IL-8 into the basolateral compartment (dotted lines show interleukin release in incubator controls). The data are presented as mean values and error bars representing 1 standard deviation of three independent experiments (ANOVA, Dunnett’s multiple comparison) with triplicate cell cultures (total *n* = 9 cell cultures). (**b**) IL-6 and (**c**) IL-8 release vs. deposited particle dose associated with positive linear correlation using standard linear regression analysis. ** *p-value* < 0.01, *** *p-value* < 0.001 compared to filter blank. **Abbreviations:** LPS = lipopolysaccharide (10 μg/mL); L = low dose; H = high dose; R^2^ = R-square linear coefficient of determination.

The release of both interleukins analyzed significantly increased in response to PM exposure (Fig 2A); IL-6 on average 1.4 fold in normal and 1.3 fold in CF HBE, as compared to field blanks (*p-values* < 0.0001), and IL-8 on average 2.2 fold in normal (*p-value* = 0.0002) and 1.6 fold in CF HBE (*p-value* < 0.0001) and irrespective of seasonal sampling. Although, the release of interleukins correlated well with the deposited dose in normal and CF HBE (R^2^ = 0.84 and 0.85 for IL-6; R^2^ = 0.73 and 0.78 for IL-8, Fig 2B–2C), there was no significant difference between PM_10_ and PM_2.5_. Exposure to the positive-control compound LPS caused the highest increases in the release of both cytokines (Fig 2A). IL-6 was on average 2.2 fold and IL-8 was 3.1 fold higher than in the field blank controls.

#### Gene expression

**Fig 3:**
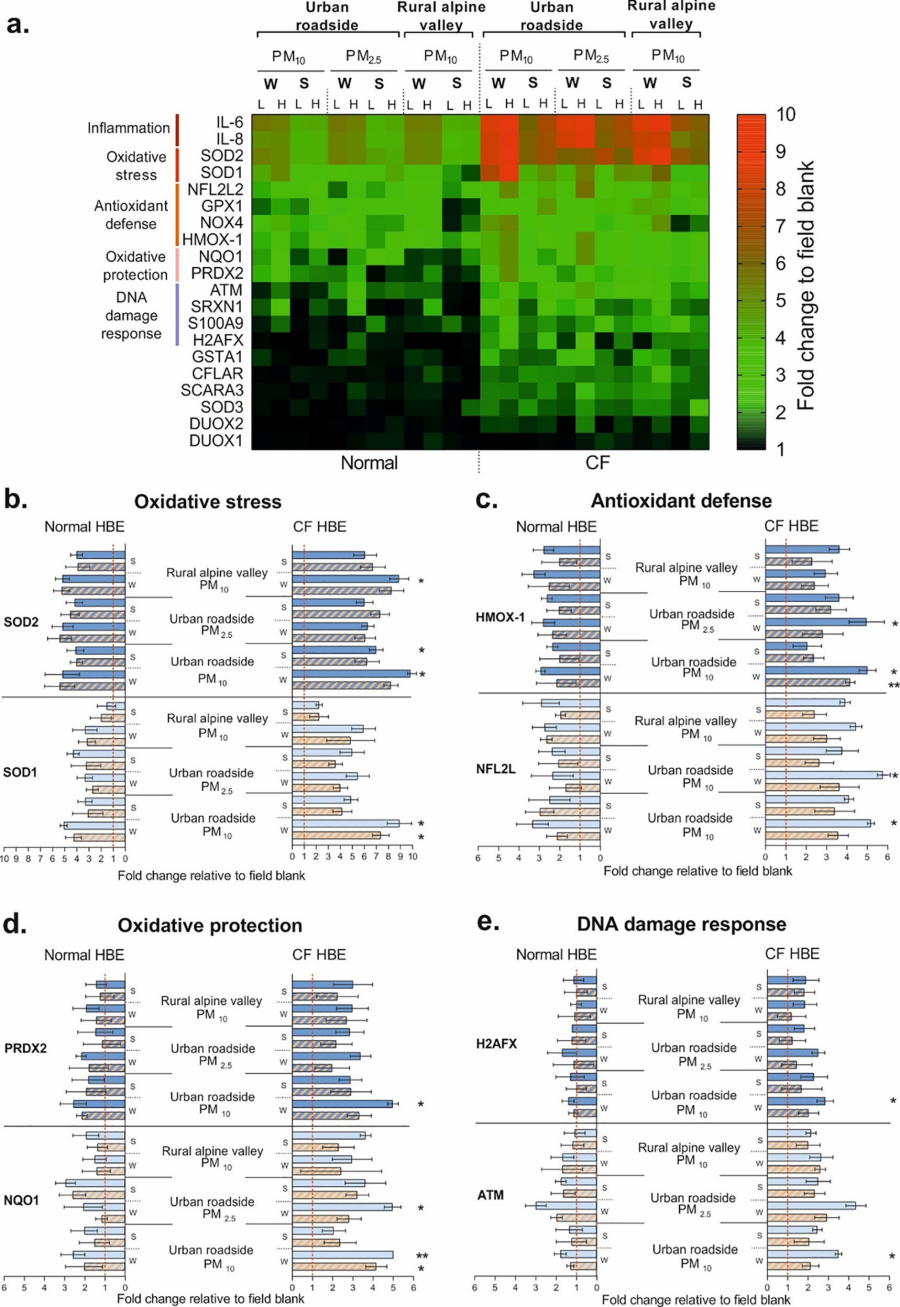
Screening of gene expression profiles and pathways associated with PM exposure in normal and CF HBE. (**a**) Gene expression profiles of the screened 20 genes, classified by PM category (PM_10_, PM_2.5_) and site (urban roadside and rural alpine valley). Fold changes of gene expressions relative to field blank controls are reported (*n* = 9). Abbreviations: IL = interleukin; GSTA1 = glutathione S-transferase alpha 1 = S100A9: S100 calcium binding protein A9; ATM = ataxia telangiectasia mutated kinase; NOX4 = NADPH oxidase 4; H2AFX = H2A histone family member X; SOD = superoxide dismutase; PRDX2 = peroxiredoxin 2; SRXN1 = sulfiredoxin 1; DUOX = dual oxidase; SCARA3 = scavenger receptor class A member 3; HMOX1 = heme oxygenase 1; NQO1 = NAD(P)H quinone dehydrogenase; GPX1 = glutathione peroxidase 1; CFLAR = CASP8 and FADD like apoptosis regulator; NFE2L2 = nuclear factor erythroid-derived 2-like 2. RNA18S5 = ribosomal RNA 18S5, used as reference gene in the unsupervised analysis. HPRT1 = hypoxanthine guanine phosphoribosyl transferase 1 and HSP90AB1 = heat shock protein 90 kDA alpha, class B, member 1 were selected as housekeeping genes. Validation of (**b**) oxidative stress, (**c**) antioxidant defense, (**d**) oxidative protection and (**e**) DNA damage response pathways in normal and CF HBE. The data are presented as mean values and standard error of the mean of three independent experiments (ANOVA, Dunnett’s multiple comparison). Filled bars represent exposure to high, banded bars to low PM dose. * *p-value* < 0.05; ** *p-value* < 0.01 referring to gene expression of CF compared to normal HBE.

Overall, the screening with gene globe array revealed common signatures of deregulated genes in both cell models, as compared to the filter blank controls. In normal HBE, the expression of 14 out of 20 genes was upregulated (seven highly and seven moderately), two were downregulated and four genes remained unchanged. In CF HBE, however, most of the selected genes (*n* = 18) were upregulated (two- to tenfold change) and only two remained unaltered (Fig 3 and S1 Fig). We further validated the upregulation of the genes with the highest degree of deregulation (superoxide dismutase 1 and 2: SOD1, SOD2; nuclear factor erythroid-derived 2-like 2: NFL2L; heme oxygenase 1: HMOX1; NAD(P)H quinone dehydrogenase: NQO1; peroxiredoxin 2: PRDX2 and ataxia telangiectasia mutated kinase: ATM) using real-time polymerase chain reaction (RT-qPCR) (Fig 3B–3E). Inflammation arising via oxidative stress response is reflected by the significantly increased expression of the oxidative stress genes SOD1 and SOD2 in both normal (*p-value* = 0.0415 for SOD1 and *p-value* = 0.0314 for SOD2) and CF HBE (*p-value* = 0.0318 and *p-value* = 0.0326) (Fig 3B). Parallel activation of the antioxidant response and of cytoprotection in normal and CF HBE is shown by upregulation of the HMOX1 (*p-value* = 0.0219, *p-value* = 0.0414), the NFL2L (*p-value* = 0.0409 and *p-value* = 0.0314), the PRDX (*p-value* = 0.0472, *p-value* = 0.0461) and the NQO1 genes (*p-value* = 0.0484, *p-value* = 0.0310) (Fig 3C and 3D). In contrast, DNA damage genes were significantly upregulated in addition to oxidative stress genes only in CF HBE (*p-value* = 0.0066 for ATM and *p-value* = 0.0452 for H2A histone family member X:H2FAFX, Fig 3E).

### Oxidant generation capacity and chemical composition of PM

#### Oxidative Potential (OP)

**Fig 4:**
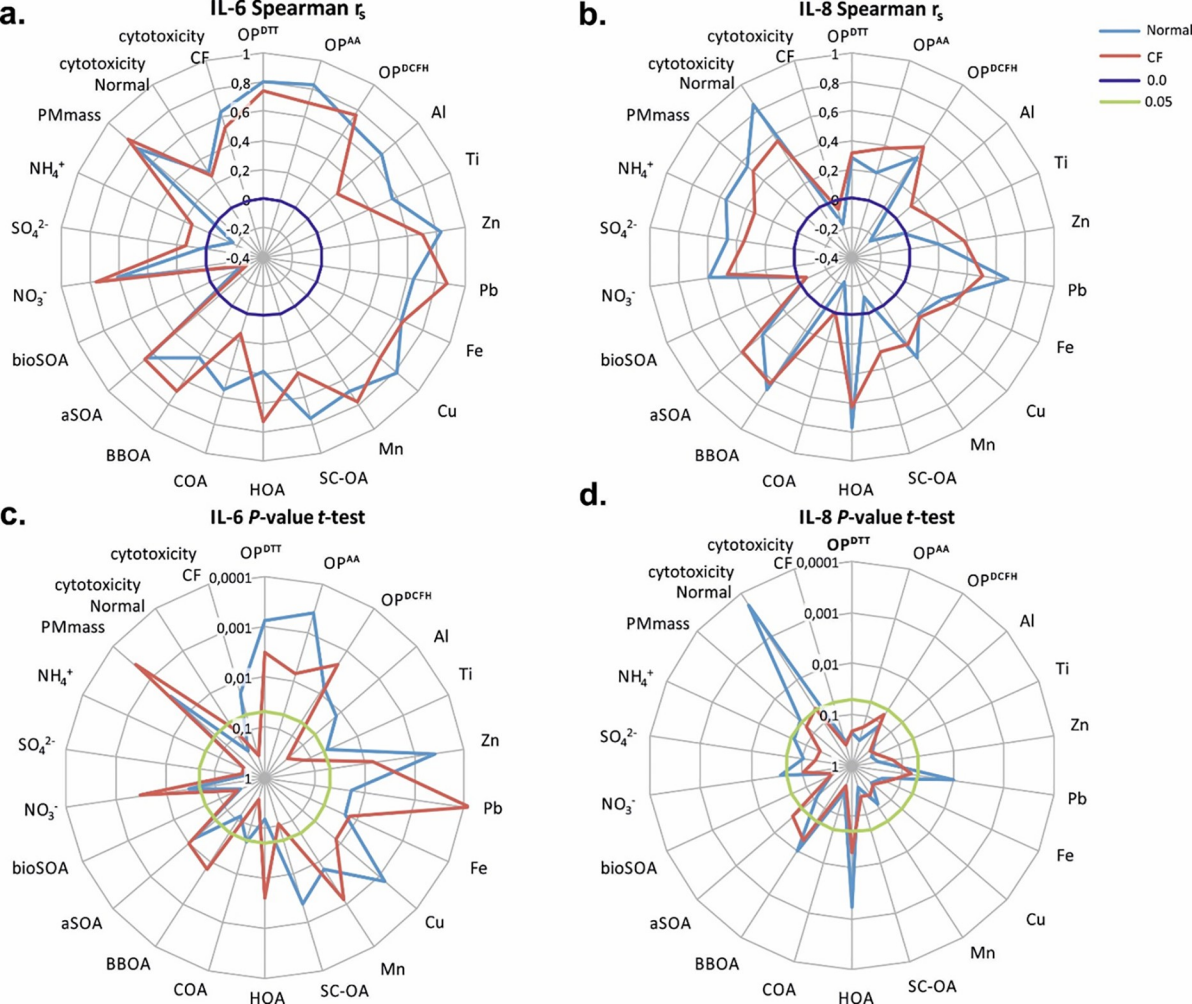
Association between cellular responses upon exposure to PM and chemical parameters of the PM. (**a**) and (**b**) non parametric correlation (Spearman *r*_*s*_), (**c**) and (**d**) Student’s *t*-test *p*-values of *r*_*s*_. The correlation was derived from the release of interleukins (IL-6 and IL-8) and lactate dehydrogenase (LDH) in normal and cystic fibrosis (CF) HBE after exposure to PM filter extracts. OP^DTT^, OP^AA^ and OP^DCFH^ refer to the oxidative potential of PM determined using the three acellular assays: dithiothreitol (DTT), ascorbic acid (AA) and 2’7’-dichlorofluorescin (DCFH). The following chemical components of PM are included (see Methods): exhaust (hydrocarbon-like OA–HOA) and non-exhaust (organic sulfur-containing OA—SCOA, metals: Cu, Fe, Mn) primary traffic emissions, cooking emissions (COA), primary biomass burning emissions (organic: BBOA, metals: Zn, Pb), crustal material (Ti, Al), anthropogenic SOA (aSOA), biogenic SOA (bioSOA) and secondary inorganic aerosol (SIA, NH_4_^+^, NO_3_^-^, SO_4_^2-^).

The assessment of the OP of PM by the acellular assays dithiothreitol (OP^DTT^), ascorbic acid (OP^AA^) and 2’7’-dichlorofluorescin (OP^DCFH^), shown in Fig 4A and 4C, revealed exposure to PM with a high OP to enhance the release of IL-6 in both cell models using the non-parametric Spearman’s rank correlation coefficient (*r*_*s*_ OP^DTT^ = 0.80, OP^AA^ = 0.82, OP^DCFH^ = 0.69 in normal and *r*_*s*_ OP^DTT^ = 0.74, OP^AA^ = 0.69, OP^DCFH^ = 0.76 in CF HBE) and thus triggering inflammation [28, 29, 40].

#### Chemical composition

To further evaluate the impact of the PM chemical composition and identify the relevant sources on the inflammatory response in HBE, we distinguished exhaust from primary traffic emissions (hydrocarbon-like OA–HOA) and non-exhaust (organic sulfur-containing OA–SCOA, metals: Cu, Fe, Mn), primary cooking emissions (COA), primary biomass burning emissions (organic: BBOA, metals: Zn, Pb), crustal material (Ti, Al), anthropogenic SOA (aSOA), biogenic SOA (bioSOA), and secondary inorganic aerosol (SIA, NH_4_^+^, NO_3_^-^, SO_4_^2-^). The results, shown in Fig 4 and Table 3, clearly demonstrate increasing IL-6 release in normal and CF HBE to relate to increasing concentrations of anthropogenic PM components.

**Table 3:** Association between cellular responses of normal and CF HBE to PM exposure and physico-chemical parameters of the PM.

Analyte	IL-6		IL-8		LDH	
	Normal	CF	Normal	CF	Normal	CF
**IL-6 Normal HBE**	**1**	0.46	0.20	0.56	0.28	0.63*
**CF HBE**	0.46	**1**	0.61*	0.46	0.50	0.15
**IL-8 Normal HBE**	0.20	0.61*	**1**	0.61*	0.84^***^	-0.16
**CF HBE**	0.56	0.46	0.61	**1**	0.54*	-0.06
**OP-DTT**	0.80^***^	0.74^**^	0.28	0.31	0.26	0.52
**OP-AA**	0.82^***^	0.69^**^	0.20	0.37	0.13	0.48
**OP-DCFH**	0.69^**^	**0.76** ^**^	0.41	0.50	0.22	0.20
**Cu**	0.80^***^	0.65*	0.20	0.22	0.29	0.69^**^
**Fe**	0.63*	0.65*	0.28	0.35	0.11	0.31
**Mn**	0.69^**^	0.78^**^	0.41	0.31	0.31	0.37
**Zn**	0.82^***^	0.69^**^	0.20	0.37	0.18	0.48
**Pb**	0.63*	0.87^***^	0.67^**^	0.50	0.52	0.15
**Al**	0.67	0.26	-0.23	0.13	-0.14	0.74^**^
**Ti**	0.56	0.39	-0.01	0.22	-0.12	0.42
**HOA**	0.38	0.73^**^	0.77^**^	0.62*	**0.59** *	-0.04
**COA**	0.54	0.14	-0.22	0.00	0.00	0.75^**^
**BBOA**	0.41	0.70^**^	0.67^**^	0.63*	0.42	-0.14
**SC-OA**	0.75^**^	0.43	-0.11	0.27	-0.11	0.57*
**aSOA**	0.65*	0.68^**^	0.41	0.58*	0.26	0.11
**bioSOA**	-0.23	-0.27	-0.05	-0.05	-0.06	0.13
**NO_3_^-^**	0.61*	0.76^**^	0.58*	0.46	0.52	0.20
**SO_4_^2-^**	0.02	0.13	0.46	0.35	0.54	0.05
**NH_4_^+^**	-0.16	0.13	0.54	0.33	0.44	-0.29
**PM Mass**	0.74^**^	0.82^***^	0.54	0.50	0.48	0.31
**LDH Normal HBE**	0.28	0.26	0.84^***^	0.54*	**1**	0.11
**CF HBE**	0.63	0.52	-0.16	-0.05	0.11	**1**

Non-parametric correlation (Spearman *r*_*s*_) determined from the release of interleukins (IL-6 and IL-8) and lactate dehydrogenase (LDH) in normal and cystic fibrosis (CF) human bronchial epithelia (HBE) 24 h after exposure to PM. The oxidative potential of PM was determined using three acellular assays: dithiothreitol (DTT), ascorbic acid (AA) and 2’7’-dichlorofluorescin (DCFH). Metals were measured by ICP-MS. Sources of organic aerosols (OA) were obtained from positive matrix factorization PMF. We resolved primary OA from non-exhaust vehicular emissions (SC-OA), traffic exhaust (hydrocarbon-like OA, HOA), cooking (COA) and biomass burning (BBOA), and secondary OA from anthropogenic (aSOA) and biogenic emissions (bioSOA).

* *p-value* < 0.05

** *p-value* < 0.01

*** *p-value* < 0.001 indicate a statistically significant correlation (*t*-test).

## Discussion

The evidence of health effects associated with source-specific PM does not indicate a clear ‘hierarchy’ of harmfulness for PM from different emission sources [2, 41]. However, results obtained suggest that a range of detrimental health effects are consistently associated with traffic-related PM, SOA, specific metals and elemental carbon in PM [5, 6, 42, 43]. Our results provide on the one hand experimental support for an increased susceptibility of persons with pre-existing pulmonary disorders to environmental PM exposure [23, 44–46]. On the other hand, we identified PM components and their biological mechanism leading to adverse effects.

By integrating information from different disciplines, the present study reveals that the major mechanism by which PM acts on the bronchial epithelium, the primarily affected lung compartment in pulmonary disease, is to proceed through an oxidant/anti-oxidant imbalance to yield inflammatory response cascades. We further show that anthropogenic PM magnifies the adverse health outcome in normal and susceptible bronchial epithelia. In addition, the increase of the deposited PM dose progressively increases epithelial damage. The four to six times enhanced cytotoxicity in CF HBE compared to normal epithelia shows that individuals with pre-existing pulmonary diseases are more susceptible to PM exposure than healthy ones.

Thus, the main driver of the adverse health effects of PM pollution is the anthropogenic PM, in particular in vulnerable individuals. Our data on the assessment of cytotoxicity and interleukin release clearly demonstrate the relation of the observed cytotoxicity to the inflammatory processes induced by PM exposure. In addition, our results on the gene expression confirm the activation of the inflammatory response pathway and reveal the upregulation of oxidative stress genes (SOD1 and SOD2) and of the antioxidant response (HMOX-1 and NFL2L) together with enhanced cytoprotection (PRDX2 and NQO1) in both cell models. Lacking activation of other genes in normal HBE indicates that the antioxidant response sufficiently protects normal epithelia. In contrast, the significantly upregulated DNA damage and oxidative stress genes in CF HBE imply that the antioxidant defense in these compromised epithelia was overcome by exposure to PM. This ultimately explains the higher cytotoxicity in CF than normal HBE.

Overall, our data clearly demonstrate that inflammation arising via oxidative stress response is reflected by significantly increased expression of the oxidative stress genes (particularly SOD2). Moreover, there is a link to the season of sample collection, mirrored by the enhanced expression of genes in samples collected in winter compared to summer in both normal and CF HBE, in particular for genes related to inflammation (IL-6 and IL-8) and antioxidant defense (SOD2).

The limited number of samples in the study, however, does not allow establishing a clear link between the observed adverse effects and the sites of PM collection or the PM category (PM_10_ samples contain the PM_2.5_ fraction). However, we provide evidence that anthropogenic sources of atmospheric PM play a more important role than biogenic emissions in inducing adverse effects to bronchial epithelia, in particular in the vulnerable population.

While considerable efforts were allocated to decrease vehicle emissions, and although PM concentrations in Europe decreased significantly during the last decades [47, 48], mitigation strategies are not equally effective in decreasing the OP of PM [49]. As outlined by Daellenbach et al. [49], due to the adoption of a renewable energy source for residential heating, the concentration of primary and aged biomass burning aerosol is expectedly rising and leads to enhanced OP [49]. Consequently, the overall health impacts of PM may increase in the near future despite the decrease in total PM mass concentrations. Our data suggest that in order to reduce the adverse health effects of ambient pollution, the commitment to effectively decrease PM mass concentration and OP has to be intensified. Specifically targeting health-relevant anthropogenic PM components (from exhaust and non-exhaust car emissions, wood combustion) is key to mitigate the adverse health impacts of PM and in particular, to protect the vulnerable population.

## Supporting information

S1 Table Calculation of the deposited particle dose in the human tracheobronchial (TB) tract at different ambient concentrations. (DOCX)

S1 Fig Screening and validation of the gene expression profile upon PM exposure in normal and CF HBE. Unsupervised hierarchical clustering analysis using the differentially expressed genes in normal and CF HBE. The heat map (Ward linkage and Euclidean distance) represents log transformed Delta Ct values. The heat map color-palette represents gene expression as indicated in the color key. Blue, green and pink lines on the left side of the heat map represent clustered genes.
(DOCX)

## References

[1] Lelieveld J. Clean air in the Anthropocene. Faraday Discuss [Internet]. 2017 8 24 [cited 2018 Aug 27];200:693–703. Available from: http://www.ncbi.nlm.nih.gov/pubmed/28702627 10.1039/c7fd90032e 28702627

[2] GBD 2016 Lifetime Risk of Stroke Collaborators, Feigin VL, Nguyen G, Cercy K, Johnson CO, Alam T, et al Global, Regional, and Country-Specific Lifetime Risks of Stroke, 1990 and 2016. N Engl J Med [Internet]. 2018 12 20 [cited 2019 Jan 23];379(25):2429–37. Available from: http://www.ncbi.nlm.nih.gov/pubmed/30575491 10.1056/NEJMoa1804492 30575491 PMC6247346

[3] GBD 2016 Risk Factors Collaborators E, Afshin A, Abajobir AA, Abate KH, Abbafati C, Abbas KM, et al Global, regional, and national comparative risk assessment of 84 behavioural, environmental and occupational, and metabolic risks or clusters of risks, 1990–2016: a systematic analysis for the Global Burden of Disease Study 2016. Lancet (London, England) [Internet]. 2017 9 16 [cited 2018 Aug 27];390(10100):1345–422. Available from: https://linkinghub.elsevier.com/retrieve/pii/S0140673617323668 10.1016/S0140-6736(17)32366-8 PMC5614451 28919119

[4] Delfino RJ, Sioutas C, Malik S. Potential role of ultrafine particles in associations between airborne particle mass and cardiovascular health. Environ Health Perspect [Internet]. 2005 8 [cited 2018 Aug 27];113(8):934–46. Available from: http://www.ncbi.nlm.nih.gov/pubmed/16079061 10.1289/ehp.7938 16079061 PMC1280331

[5] Morakinyo OM, Mokgobu MI, Mukhola MS, Hunter RP. Health Outcomes of Exposure to Biological and Chemical Components of Inhalable and Respirable Particulate Matter. Int J Environ Res Public Health [Internet]. 2016 6 14 [cited 2018 Aug 27];13(6):592 Available from: http://www.mdpi.com/1660-4601/13/6/592 10.3390/ijerph13060592 PMC4924049 27314370

[6] Bates JT, Weber RJ, Abrams J, Verma V, Fang T, Klein M, et al Reactive Oxygen Species Generation Linked to Sources of Atmospheric Particulate Matter and Cardiorespiratory Effects. Environ Sci Technol [Internet]. 2015 11 17 [cited 2018 Aug 27];49(22):13605–12. Available from: http://www.ncbi.nlm.nih.gov/pubmed/26457347 10.1021/acs.est.5b02967 26457347

[7] Hooper LG, Kaufman JD. Ambient Air Pollution and Clinical Implications for Susceptible Populations. Ann Am Thorac Soc [Internet]. 2018 4 [cited 2019 Jan 9];15(Supplement_2):S64–8. Available from: http://www.ncbi.nlm.nih.gov/pubmed/29676646 29676646 10.1513/AnnalsATS.201707-574MG PMC5955035

[8] Delfino RJ, Staimer N, Tjoa T, Gillen DL, Schauer JJ, Shafer MM. Airway inflammation and oxidative potential of air pollutant particles in a pediatric asthma panel. J Expo Sci Environ Epidemiol [Internet]. 2013 9 15 [cited 2019 Jan 23];23(5):466–73. Available from: http://www.nature.com/articles/jes201325 10.1038/jes.2013.25 23673461 PMC4181605

[9] Li N, Hao M, Phalen RF, Hinds WC, Nel AE. Particulate air pollutants and asthma. A paradigm for the role of oxidative stress in PM-induced adverse health effects. Clin Immunol [Internet]. 2003 12 [cited 2019 Jan 23];109(3):250–65. Available from: http://www.ncbi.nlm.nih.gov/pubmed/14697739 10.1016/j.clim.2003.08.006 14697739

[10] Xiao GG, Wang M, Li N, Loo JA, Nel AE. Use of proteomics to demonstrate a hierarchical oxidative stress response to diesel exhaust particle chemicals in a macrophage cell line. J Biol Chem [Internet]. 2003 12 12 [cited 2019 Apr 2];278(50):50781–90. Available from: http://www.jbc.org/lookup/doi/10.1074/jbc.M306423200 14522998 10.1074/jbc.M306423200

[11] Van Den Heuvel R, Den Hond E, Govarts E, Colles A, Koppen G, Staelens J, et al Identification of PM 10 characteristics involved in cellular responses in human bronchial epithelial cells (Beas-2B). Environ Res [Internet]. 2016 8 [cited 2018 Aug 27];149:48–56. Available from: http://www.ncbi.nlm.nih.gov/pubmed/27177354 10.1016/j.envres.2016.04.029 27177354

[12] Ayres JG, Borm P, Cassee FR, Castranova V, Donaldson K, Ghio A, et al Evaluating the toxicity of airborne particulate matter and nanoparticles by measuring oxidative stress potential—a workshop report and consensus statement. Inhal Toxicol [Internet]. 2008 1 6 [cited 2019 Jan 23];20(1):75–99. Available from: http://www.tandfonline.com/doi/full/10.1080/08958370701665517 10.1080/08958370701665517 18236225

[13] Usemann J, Roth M, Bisig C, Comte P, Czerwinski J, Mayer ACR, et al Gasoline particle filter reduces oxidative DNA damage in bronchial epithelial cells after whole gasoline exhaust exposure in vitro. Sci Rep [Internet]. 2018 12 2 [cited 2018 Aug 27];8(1):2297 Available from: http://www.ncbi.nlm.nih.gov/pubmed/29396482 10.1038/s41598-018-20736-z 29396482 PMC5797118

[14] Oberdörster G, Maynard A, Donaldson K, Castranova V, Fitzpatrick J, Ausman K, et al Principles for characterizing the potential human health effects from exposure to nanomaterials: elements of a screening strategy. Part Fibre Toxicol [Internet]. 2005 10 6 [cited 2019 Jan 23];2(1):8 Available from: http://www.ncbi.nlm.nih.gov/pubmed/16209704 16209704 10.1186/1743-8977-2-8 PMC1260029

[15] Bates JT, Fang T, Verma V, Zeng L, Weber RJ, Tolbert PE, et al Review of Acellular Assays of Ambient Particulate Matter Oxidative Potential: Methods and Relationships with Composition, Sources, and Health Effects. Environ Sci Technol [Internet]. 2019 4 3 [cited 2019 Apr 12];acs.est.8b03430 Available from: http://pubs.acs.org/doi/10.1021/acs.est.8b03430 10.1021/acs.est.8b03430 30830764

[16] Calas A, Uzu G, Martins JMF, Voisin D, Spadini L, Lacroix T, et al The importance of simulated lung fluid (SLF) extractions for a more relevant evaluation of the oxidative potential of particulate matter. Sci Rep [Internet]. 2017 12 14 [cited 2018 Aug 27];7(1):11617 Available from: http://www.ncbi.nlm.nih.gov/pubmed/28912590 10.1038/s41598-017-11979-3 28912590 PMC5599505

[17] Barmpadimos I, Hueglin C, Keller J, Henne S, Prévôt ASH. Influence of meteorology on PM _10_ trends and variability in Switzerland from 1991 to 2008. Atmos Chem Phys [Internet]. 2011 2 28 [cited 2020 Jan 7];11(4):1813–35. Available from: https://www.atmos-chem-phys.net/11/1813/2011/

[18] Verma V, Rico-Martinez R, Kotra N, King L, Liu J, Snell TW, et al Contribution of Water-Soluble and Insoluble Components and Their Hydrophobic/Hydrophilic Subfractions to the Reactive Oxygen Species-Generating Potential of Fine Ambient Aerosols. Environ Sci Technol [Internet]. 2012 10 16 [cited 2019 Aug 7];46(20):11384–92. Available from: http://www.ncbi.nlm.nih.gov/pubmed/22974103 10.1021/es302484r 22974103

[19] Daellenbach KR, Bozzetti C, Křepelová A, Canonaco F, Wolf R, Zotter P, et al Characterization and source apportionment of organic aerosol using offline aerosol mass spectrometry. Atmos Meas Tech [Internet]. 2016 1 15 [cited 2019 Jan 29];9(1):23–39. Available from: https://www.atmos-meas-tech.net/9/23/2016/

[20] Pillai DK, Sankoorikal B-J V, Johnson E, Seneviratne AN, Zurko J, Brown KJ, et al Directional secretomes reflect polarity-specific functions in an in vitro model of human bronchial epithelium. Am J Respir Cell Mol Biol [Internet]. 2014 2 6 [cited 2018 Aug 27];50(2):292–300. Available from: http://www.atsjournals.org/doi/abs/10.1165/rcmb.2013-0188OC 24010916 10.1165/rcmb.2013-0188OC PMC3930950

[21] Peters-Hall JR, Brown KJ, Pillai DK, Tomney A, Garvin LM, Wu X, et al Quantitative Proteomics Reveals an Altered Cystic Fibrosis *In Vitro* Bronchial Epithelial Secretome. Am J Respir Cell Mol Biol [Internet]. 2015 7 [cited 2018 Aug 27];53(1):22–32. Available from: http://www.ncbi.nlm.nih.gov/pubmed/25692303 10.1165/rcmb.2014-0256RC 25692303 PMC4566109

[22] Krapf M, Künzi L, Allenbach S, Bruns EA, Gavarini I, El-Haddad I, et al Wood combustion particles induce adverse effects to normal and diseased airway epithelia. Environ Sci Process Impacts [Internet]. 2017 4 19 [cited 2018 Aug 27];19(4):538–48. Available from: http://www.ncbi.nlm.nih.gov/pubmed/28239691 10.1039/c6em00586a 28239691

[23] Künzi L, Krapf M, Daher N, Dommen J, Jeannet N, Schneider S, et al Toxicity of aged gasoline exhaust particles to normal and diseased airway epithelia. Sci Rep [Internet]. 2015 12 29 [cited 2018 Aug 27];5(1):11801 Available from: http://www.ncbi.nlm.nih.gov/pubmed/26119831 26119831 10.1038/srep11801 PMC4484354

[24] Fulcher ML, Gabriel S, Burns K a, Yankaskas JR, Randell SH. Well-differentiated human airway epithelial cell cultures. Methods Mol Med. 2005;107:183–206. 10.1385/1-59259-861-7:183 15492373

[25] Kim MD, Baumlin N, Yoshida M, Polineni D, Salathe SF, David JK, et al Losartan Rescues Inflammation-Related Mucociliary Dysfunction in Relevant Models of Cystic Fibrosis. Am J Respir Crit Care Med [Internet]. 2019 10 15 [cited 2020 Jan 7]; Available from: http://www.ncbi.nlm.nih.gov/pubmed/31613648 10.1164/rccm.201905-0990OC PMC6999107 31613648

[26] Canonaco F, Crippa M, Slowik JG, Baltensperger U, Prévôt ASH. SoFi, an IGOR-based interface for the efficient use of the generalized multilinear engine (ME-2) for the source apportionment: ME-2 application to aerosol mass spectrometer data. Atmos Meas Tech [Internet]. 2013 12 23 [cited 2019 Jan 29];6(12):3649–61. Available from: https://www.atmos-meas-tech.net/6/3649/2013/

[27] Paatero P. The Multilinear Engine—A Table-Driven, Least Squares Program for Solving Multilinear Problems, Including the *n* -Way Parallel Factor Analysis Model. J Comput Graph Stat [Internet]. 1999 12 [cited 2019 Jan 29];8(4):854–88. Available from: http://www.tandfonline.com/doi/abs/10.1080/10618600.1999.10474853

[28] Vlachou A, Daellenbach KR, Bozzetti C, Chazeau B, Salazar GA, Szidat S, et al Advanced source apportionment of carbonaceous aerosols by coupling offline AMS and radiocarbon size-segregated measurements over a nearly 2-year period. Atmos Chem Phys [Internet]. 2018 5 3 [cited 2019 Jan 29];18(9):6187–206. Available from: https://www.atmos-chem-phys.net/18/6187/2018/

[29] Daellenbach KR, Stefenelli G, Bozzetti C, Vlachou A, Fermo P, Gonzalez R, et al Long-term chemical analysis and organic aerosol source apportionment at nine sites in central Europe: source identification and uncertainty assessment. Atmos Chem Phys [Internet]. 2017 11 8 [cited 2019 Jan 29];17(21):13265–82. Available from: https://www.atmos-chem-phys.net/17/13265/2017/

[30] Birch ME, Cary RA. Elemental Carbon-Based Method for Monitoring Occupational Exposures to Particulate Diesel Exhaust. Aerosol Sci Technol [Internet]. 1996 1 [cited 2019 Jan 29];25(3):221–41. Available from: http://www.tandfonline.com/doi/abs/10.1080/02786829608965393

[31] Cavalli F, Viana M, Yttri KE, Genberg J, Putaud J-P. Toward a standardised thermal-optical protocol for measuring atmospheric organic and elemental carbon: the EUSAAR protocol. Atmos Meas Tech [Internet]. 2010 1 26 [cited 2019 Jan 29];3(1):79–89. Available from: http://www.atmos-meas-tech.net/3/79/2010/

[32] Piot C, Jaffrezo J-L, Cozic J, Pissot N, El Haddad I, Marchand N, et al Quantification of levoglucosan and its isomers by High Performance Liquid Chromatography &amp;ndash; Electrospray Ionization tandem Mass Spectrometry and its applications to atmospheric and soil samples. Atmos Meas Tech [Internet]. 2012 1 12 [cited 2019 Jan 29];5(1):141–8. Available from: https://www.atmos-meas-tech.net/5/141/2012/

[33] Jaffrezo J., Calas N, Bouchet M. Carboxylic acids measurements with ionic chromatography. Atmos Environ [Internet]. 1998 8 1 [cited 2019 Jan 29];32(14–15):2705–8. Available from: https://www.sciencedirect.com/science/article/pii/S1352231098000260

[34] Waked A, Favez O, Alleman LY, Piot C, Petit J-E, Delaunay T, et al Atmospheric Chemistry and Physics Source apportionment of PM 10 in a north-western Europe regional urban background site (Lens, France) using positive matrix factorization and including primary biogenic emissions. Atmos Chem Phys [Internet]. 2014 [cited 2019 Jan 29];14:3325–46. Available from: www.atmos-chem-phys.net/14/3325/2014/

[35] Calas A, Uzu G, Kelly FJ, Houdier S, Martins JMF, Thomas F, et al Comparison between five acellular oxidative potential measurement assays performed with detailed chemistry on PM&lt;sub&gt;10&lt;/sub&gt; samples from the city of Chamonix (France). Atmos Chem Phys [Internet]. 2018 6 5 [cited 2019 Jan 29];18(11):7863–75. Available from: https://www.atmos-chem-phys.net/18/7863/2018/

[36] Foucaud L, Wilson MR, Brown DM, Stone V. Measurement of reactive species production by nanoparticles prepared in biologically relevant media. Toxicol Lett [Internet]. 2007 11 1 [cited 2019 Jan 29];174(1–3):1–9. Available from: https://linkinghub.elsevier.com/retrieve/pii/S0378427407008296 10.1016/j.toxlet.2007.08.001 17888595

[37] Miller FJ, Asgharian B, Schroeter JD, Price O. Improvements and additions to the Multiple Path Particle Dosimetry model. J Aerosol Sci [Internet]. 2016 9 1 [cited 2019 Jan 14];99:14–26. Available from: https://www.sciencedirect.com/science/article/pii/S0021850215300860?via%3Dihub

[38] Zheng Y, Xue T, Zhang Q, Geng G, Tong D, Li X, et al Air quality improvements and health benefits from China’s clean air action since 2013. Environ Res Lett [Internet]. 2017 11 1;12(11):114020 Available from: http://stacks.iop.org/1748-9326/12/i=11/a=114020?key=crossref.a5e68850f6814639c86a86dbeb46f784

[39] van der Kamp J, Bachmann TM. Health-Related External Cost Assessment in Europe: Methodological Developments from ExternE to the 2013 Clean Air Policy Package. Environ Sci Technol [Internet]. 2015 3 3 [cited 2019 Jan 14];49(5):2929–38. Available from: http://www.ncbi.nlm.nih.gov/pubmed/25664763 10.1021/es5054607 25664763

[40] Daellenbach KR, Kourtchev I, Vogel AL, Bruns EA, Jiang J, Petäjä T, et al Impact of anthropogenic and biogenic sources on the seasonal variation in the molecular composition of urban organic aerosols: a field and laboratory study using ultra-high-resolution mass spectrometry. Atmos Chem Phys [Internet]. 2019 5 7 [cited 2020 Jan 7];19(9):5973–91. Available from: https://www.atmos-chem-phys.net/19/5973/2019/

[41] Cassee FR, Héroux M-E, Gerlofs-Nijland ME, Kelly FJ. Particulate matter beyond mass: recent health evidence on the role of fractions, chemical constituents and sources of emission. Inhal Toxicol [Internet]. 2013 12 4 [cited 2019 Apr 12];25(14):802–12. Available from: http://www.tandfonline.com/doi/full/10.3109/08958378.2013.850127 24304307 10.3109/08958378.2013.850127 PMC3886392

[42] McDonald JD, Doyle-Eisele M, Kracko D, Lund A, Surratt JD, Hersey SP, et al Cardiopulmonary response to inhalation of secondary organic aerosol derived from gas-phase oxidation of toluene. Inhal Toxicol [Internet]. 2012 9 6 [cited 2019 Apr 12];24(11):689–97. Available from: http://www.tandfonline.com/doi/full/10.3109/08958378.2012.712164 22954394 10.3109/08958378.2012.712164

[43] Chen LC, Lippmann M. Effects of Metals within Ambient Air Particulate Matter (PM) on Human Health. Inhal Toxicol [Internet]. 2009 1 [cited 2019 Apr 12];21(1):1–31. Available from: http://www.ncbi.nlm.nih.gov/pubmed/18803063 10.1080/08958370802105405 18803063

[44] Goss CH, Newsom SA, Schildcrout JS, Sheppard L, Kaufman JD. Effect of ambient air pollution on pulmonary exacerbations and lung function in cystic fibrosis. Am J Respir Crit Care Med [Internet]. 2004 4 1 [cited 2019 Apr 10];169(7):816–21. Available from: http://www.atsjournals.org/doi/abs/10.1164/rccm.200306-779OC 14718248 10.1164/rccm.200306-779OC

[45] Rückerl R, Schneider A, Breitner S, Cyrys J, Peters A. Health effects of particulate air pollution: A review of epidemiological evidence. Inhal Toxicol [Internet]. 2011 8 25 [cited 2019 Apr 10];23(10):555–92. Available from: http://www.tandfonline.com/doi/full/10.3109/08958378.2011.593587 21864219 10.3109/08958378.2011.593587

[46] Sacks JD, Stanek LW, Luben TJ, Johns DO, Buckley BJ, Brown JS, et al Particulate Matter–Induced Health Effects: Who Is Susceptible? Environ Health Perspect [Internet]. 2011 4 [cited 2019 Apr 12];119(4):446–54. Available from: http://www.ncbi.nlm.nih.gov/pubmed/20961824 10.1289/ehp.1002255 20961824 PMC3080924

[47] EEA. European Environment Agency: Air quality in Europe—2018. No Title [Internet]. 2018. Available from: https://www.eea.europa.eu/publications/air-quality-in-europe-2018

[48] Tørseth K, Aas W, Breivik K, Fjæraa AM, Fiebig M, Hjellbrekke AG, et al Introduction to the European Monitoring and Evaluation Programme (EMEP) and observed atmospheric composition change during 1972&amp;ndash;2009. Atmos Chem Phys [Internet]. 2012 6 22 [cited 2019 Apr 2];12(12):5447–81. Available from: https://www.atmos-chem-phys.net/12/5447/2012/

[49] Daellenbach K. R., Uzu G., Jiang J., Cassagnes L.-E., Leni Z., Vlachou A., et al The sources of harmful components in particulare air pollution in Europe. Nature, Press 2020 2020. 10.1038/s41586-020-2902-8 33208962

