# Supplementary material for: Oxidative stress-induced inflammation in susceptible airways by anthropogenic aerosol
Source: PLoS One. 2020 Nov 18;15(11):e0233425. doi: 10.1371/journal.pone.0233425 (PMC7673561; doi:10.1371/journal.pone.0233425)
Supplement: S1 Table — (DOCX) [file pone.0233425.s001.docx]

**S1 Table. Calculation of the deposited particle dose in the human tracheobronchial (TB) tract at different ambient concentrations.**

| Particle size, diameter [nm] | | 250 |  |  |  |
| --- | --- | --- | --- | --- | --- |
| Tidal Volume, *V*_T_ [m^3^] | | 0.000625 |  |  |  |
| Breathing frequency, *f* [min^-1^] | | 12 |  |  |  |
| Inhaled air volume/h (adult) [m^3^] | | 0.45 |  |  |  |
| Inhaled air volume/24h (adult) [m^3^] | | 10.8 |  |  |  |
| Surface area [cm^2^] | | 2471 |  |  |  |
| Deposition efficiency | | 0.066 |  | | |
| PM dose applied [µg/cm^2^] | |  | **Low** 0.93 | **Average** 7.70 | **High** 18.80 |
| Mass conc. [µg/m^3^] | **20** |  |  |  |  |
| Mass/surface area [ng/cm^2^] / time | | 6 / 1 d | 928 / 160 d | 7621 / 3.6 y | 18800 / 8.8 y |
|  |  |  |  |  |  |
| Mass conc. [µg/m^3^] | **100** |  |  |  |  |
| Mass/surface area [ng/cm^2^] / time | | 29 / 1 d | 870 / 30 d | 7410 / 265 d | 18790 / 1.7 y |
|  |  |  |  |  |  |
| Mass conc. [µg/m^3^] | **500** |  |  |  |  |
| Mass/surface area [ng/cm^2^] / time | | 144 / 1 d | 1008 / 7 d | 7410 / 65 d | 18720 / 130 d |
|  |  |  |  |  |  |
| Mass conc. [µg/m^3^] | **1000** |  |  |  |  |
| Mass/surface area [ng/cm^2^] / time | | 288 / 1 d | 864 / 3 d | 7200 / 25 d | 18720 / 65 d |
| Deposition of particles in the tracheobronchial region upon inhalation using the Multiple Path Particle Dosimetry model (MPPD v 3.04) [37]. Abbreviations: d = days, y = years | | | | | |
