# Supplementary material for: Oxidative stress-induced inflammation in susceptible airways by anthropogenic aerosol
Source: PLoS One. 2020 Nov 18;15(11):e0233425. doi: 10.1371/journal.pone.0233425 (PMC7673561; doi:10.1371/journal.pone.0233425)
Supplement: S1 Fig — Unsupervised hierarchical clustering analysis using the differentially expressed genes in normal and CF HBE. The heat map (Ward linkage and Euclidean distance) represents log transformed Delta Ct values. The heat map color-palette represents gene expression as indicated in the color key. Blue, green and pink lines on the left side of the heat map represent clustered genes. (DOCX) [file pone.0233425.s002.docx]

**
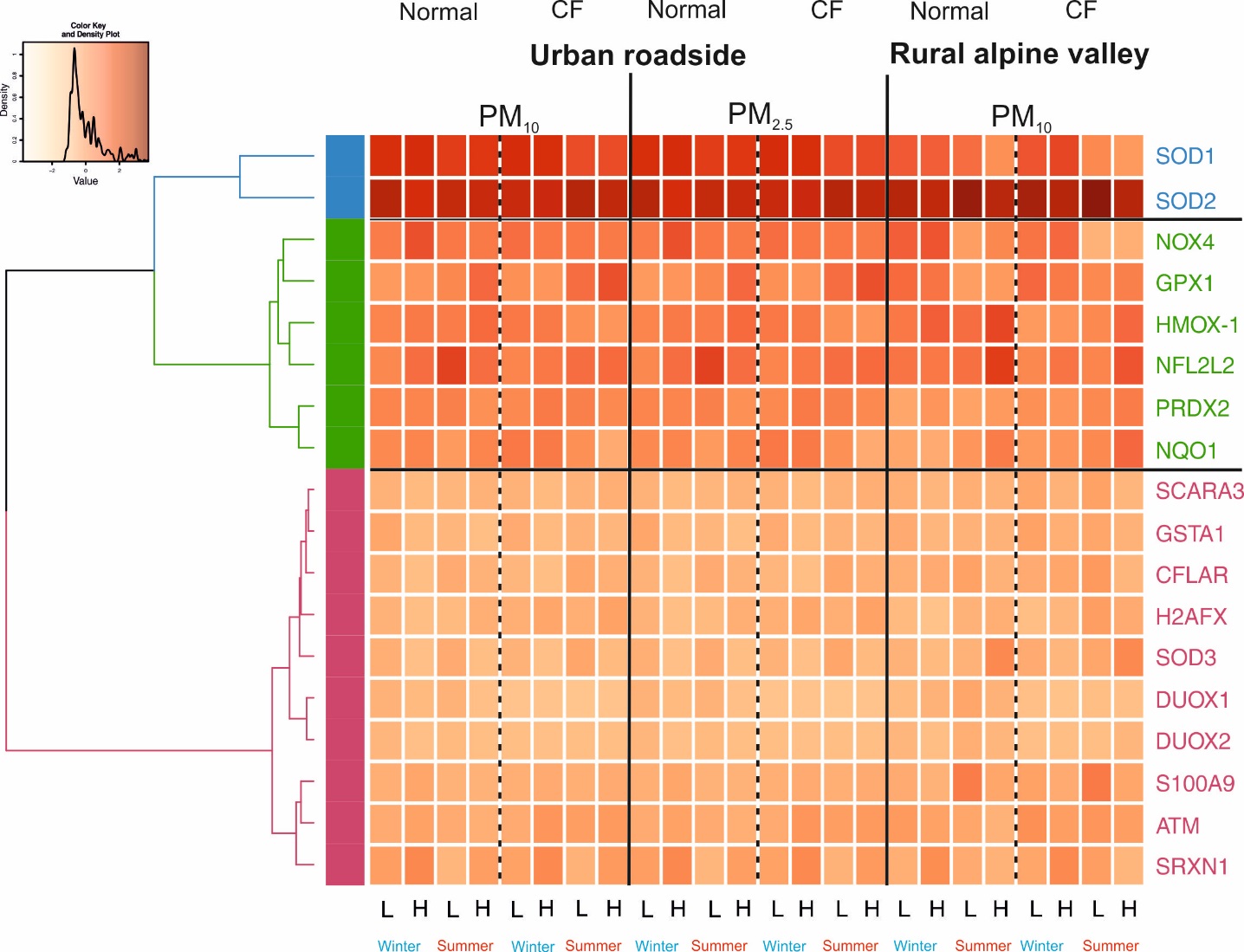
**

**S1 Fig. Screening and validation of the gene expression profile upon PM exposure in normal and CF HBE.** Unsupervised hierarchical clustering analysis using the differentially expressed genes in normal and CF HBE. The heat map (Ward linkage and Euclidean distance) represents log transformed Delta Ct values. The heat map color-palette represents gene expression as indicated in the color key. Blue, green and pink lines on the left side of the heat map represent clustered genes.
